# Novel Use of Vitamin B2 as a Fluorescent Tracer in Aerosol and Droplet Contamination Models in Otolaryngology

**DOI:** 10.1177/0003489420949588

**Published:** 2020-08-14

**Authors:** Edward S. Sim, Harish Dharmarajan, Devi Sai Sri Kavya Boorgu, Lindsey Goyal, Michael Weinstock, Rachel Whelan, Monika E. Freiser, Timothy E. Corcoran, Noel Jabbour, Eric Wang, David H. Chi

**Affiliations:** 1 University of Pittsburgh School of Medicine, University of Pittsburgh, Pittsburgh, PA, USA; 2 Department of Otolaryngology, University of Pittsburgh Medical Center, Pittsburgh, PA, USA; 3 Division of Pulmonary, Allergy and Critical Care Medicine, University of Pittsburgh Medical Center, Pittsburgh, PA, USA

**Keywords:** otolaryngology, COVID-19, aerosol, aerodynamics, contamination, impactor

## Abstract

**Objective:**

During the COVID-19 era, a reliable method for tracing aerosols and droplets generated during otolaryngology procedures is needed to accurately assess contamination risk and to develop mitigation measures. Prior studies have not investigated the reliability of different fluorescent tracers for the purpose of studying aerosols and small droplets. Objectives include (1) comparing vitamin B2, fluorescein, and a commercial fluorescent green dye in terms of particle dispersion pattern, suspension into aerosols and small droplets, and fluorescence in aerosolized form and (2) determining the utility of vitamin B2 as a fluorescent tracer coating the aerodigestive tract mucosa in otolaryngology contamination models.

**Methods:**

Vitamin B2, fluorescein, and a commercial fluorescent dye were aerosolized using a nebulizer and passed through the nasal cavity from the trachea in a retrograde-intubated cadaveric head. In another scenario, vitamin B2 was irrigated to coat the nasal cavity and nasopharyngeal mucosa of a cadaveric head for assessment of aerosol and droplet generation from endonasal drilling. A cascade impactor was used to collect aerosols and small droplets ≤14.1 µm based on average aerodynamic diameter, and the collection chambers were visualized under UV light.

**Results:**

When vitamin B2 was nebulized, aerosols ≤5.4 µm were generated and the collected particles were fluorescent. When fluorescein and the commercial water tracer dye were nebulized, aerosols ≤8.61 µm and ≤2.08 µm respectively were generated, but the collected aerosols did not appear visibly fluorescent. Endonasal drilling in the nasopharynx coated with vitamin B2 irrigation yielded aerosols ≤3.30 µm that were fluorescent under UV light.

**Conclusion:**

Vitamin B2’s reliability as a fluorescent tracer when suspended in aerosols and small droplets ≤14.1 µm and known mucosal safety profile make it an ideal compound compared to fluorescein and commercial water-based fluorescent dyes for use as a safe fluorescent tracer in healthcare contamination models especially with human subjects.

## Introduction

Due to the global COVID-19 pandemic, there has been an increased interest in studying the aerodynamic properties of aerosols and droplets. There is uncertainty whether aerosols may harbor enough viable SARS-CoV-2 virus to cause infection; a comprehensive understanding of aerosol and droplet behavior and mitigation strategies are needed in order to effectively limit infectious spread.^1^ Otolaryngologists, in particular, are at high-risk of nosocomial SARS-CoV-2 infections due to the numerous aerosol and droplet generating procedures involving manipulation of potentially high viral load tissue. Distinctly characterizing aerosols and droplets based on aerodynamic diameter is critical to assessing risk of contamination due to the varying routes of transmission and infectious potential of viruses suspended in different sized particles. In this study, aerosols are defined as particles with an aerodynamic diameter ≤10 µm whereas droplets are characterized as those with diameters >10 µm. This is in accordance with differences in particle dispersion and deposition into upper and lower airways.^2^ Multiple fluorescent tracers such as Glo Germ (Glo Germ Company, Moab, Utah) and fluorescein have been used in healthcare contamination models.^3,4^ For risk assessment of otolaryngology procedures, fluorescein has been the primary tracer utilized.^5-7^ However, these tracers are limited due to their potential toxicity in humans and unknown capacity to fluoresce in an aerosolized form. Riboflavin, vitamin B2, is a water-soluble vitamin that exhibits fluorescence under UV light due to the π conjugation and resonance present in its chemical structure.^8^ Riboflavin’s fluorescent properties have allowed for its use as a tracer in many different applications. In this study, we evaluate the utility of riboflavin, vitamin B2, as a fluorescent aerosol tracer, compare its properties to conventional fluorescent tracers, and explore its potential applications in human studies.

## Materials and Methods

**Figure 1.**
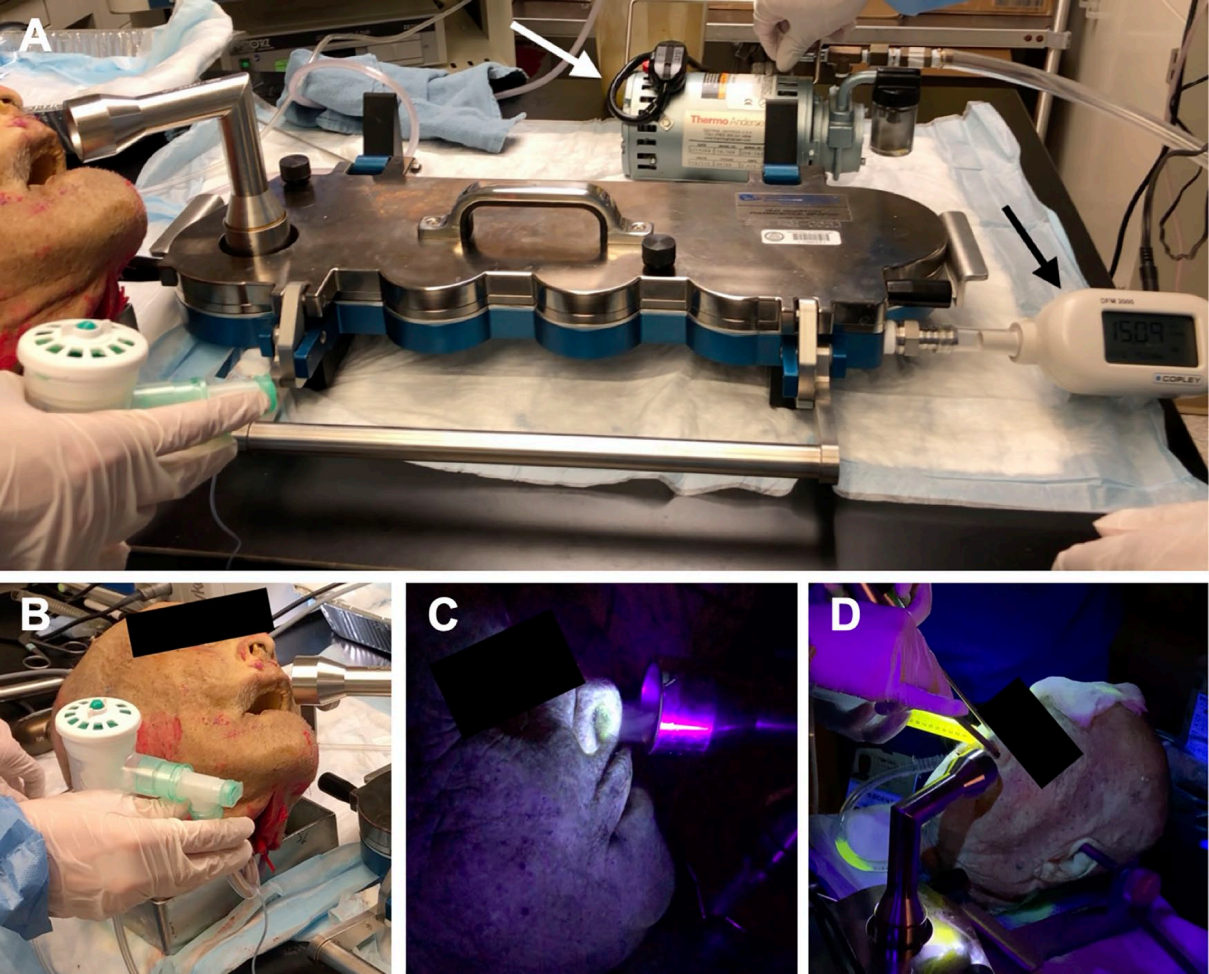
Setup of trials. (A) Impactor setup with vacuum generator (white arrow) and flow meter (black arrow). (B) Breath Actuated Nebulizer system attached to retrograded 8.0 ETT in cadaver trachea. (C) Positioning of cadaveric nares adjacent to impactor inlet nozzle. Aerosols plumes can be visualized exiting nares under UV light and entering impactor inlet nozzle. (D) Setup of endonasal drilling trial with vitamin B2 irrigation.

This study was conducted with approval from the University of Pittsburgh Committee for Oversight of Research and Clinical Training Involving Decedents (CORID #1054). Vitamin B2 1 g/L in 0.9% normal saline, fluorescein 1 g/L in 0.9% normal saline, and a stock solution of a commercially available green fluorescent water tracing dye (Factor Direct Chemicals, Copiague, New York) were used as reagents. Aerosols of these solutions were generated using an AeroEclipse II Breath Actuated Nebulizer (BAN) (Trudell Medical International, London, ON). The BAN system was used to create aerosol particles with an average mass median aerodynamic diameter (MMAD) of 2.8 µm.^9,10^ The trachea of an adult cadaver head was intubated retrograde with an 8.0 ETT with the distal tip projecting into the nasopharynx and confirmed with an endoscope. The BAN nebulizer was attached to the proximal end of the ETT and generated aerosols that traveled through the ETT and exited through the nares (Figure 1B). Aerosol plumes could be visualized under room light and were accentuated under UV blacklight when fluorescent solutions were used (Supplemental Video).

A Model 170 Next Generation Impactor™ (NGI™) was used to collect particles into 8 stages with the following aerodynamic diameters (D_50_, cutoff diameter at 50% collection efficiency): 14.1 µm, 8.61 µm, 5.39 µm, 3.30 µm, 2.08 µm, 1.36 µm, 0.98 µm, and less than 0.98 µm.^11,12^ Each impactor stage reflects an average particle aerodynamic diameter given an input flow rate of 15 L/min (Figure 1A). This size range characterizes aerosols that are likely to reach the infraglottic airway (<10 µm) and settle into lung alveoli (<5 µm).^2^ Each stage has a corresponding capture chamber that was lined with a removable piece of aluminum foil. The collected particulate matter on each foil was visualized under UV light following each trial. The nares of the cadaveric head were placed directly adjacent to the NGI™ input nozzle (Figure 1C). Each trial lasted 2 minutes and included using the following solutions via the BAN system to generate aerosols: 0.9% normal saline, 1 g/L riboflavin (vitamin B2), 1 g/L fluorescein and a stock solution of a green fluorescent water tracer (Factor Direct Chemicals, Copiague, New York). We also performed a trial of endonasal drilling in a cadaveric head with a coarse diamond burr (Stryker©, Kalamazoo, MI) using 1 g/L vitamin B2 solution for irrigation; in this scenario, vitamin B2 irrigation was used to coat the nasal cavity and nasopharyngeal mucosa prior to drilling and intermittently while drilling occurred (Figure 1D). A UV flashlight was used to visualize the filtrate at each stage in a dark room. The impactor was used to determine the aerodynamic size distribution of the aerosol and small droplet particles generated from each simulation scenario.

## Results

**Figure 2.**
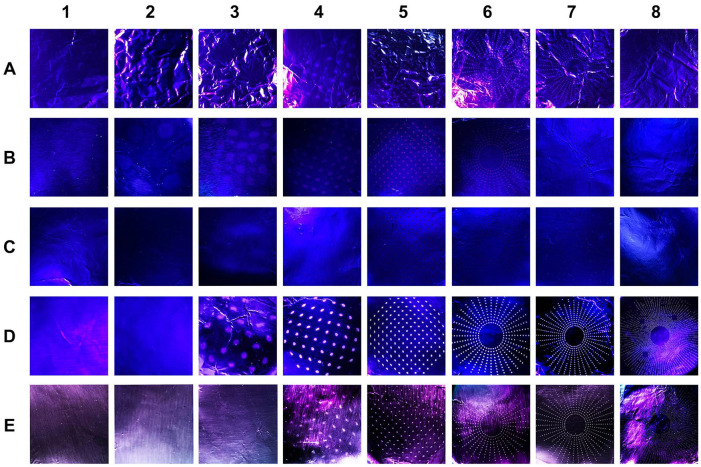
Photographs of particulates collected from impactor trials. Photographs of removable foil pieces that lined the cascade impactor capture chambers illuminated under UV light for trials using nebulized saline (negative control, A), nebulized fluorescein (B), nebulized green water tracer dye (C), nebulized vitamin B2 (D), and coarse diamond drilling of cadaver head with Vitamin B2 irrigation (E). Particles filtered based on average aerodynamic diameter into 8 impactor stages - (1) 14.1 µm, (2) 8.61 µm, (3) 5.39 µm, (4) 3.30 µm, (5) 2.08 µm, (6) 1.36 µm, (7) 0.98 µm, and (8) <0.98 μm – are displayed.

The vitamin B2, fluorescein, and commercial green tracer dye exhibited baseline fluorescence in solution form. As a negative control, the BAN system was loaded with 0.9% normal saline to ensure that the saline solution did not exhibit fluorescence and that the BAN system could effectively produce aerosols detectable by the impactor. For this trial, aerosols approximately 3.30 µm or smaller were generated, but the small droplets did not appear fluorescent under UV light (Figure 2A; Table 1). The 1 g/L fluorescein solution generated aerosols ≤8.61 µm. The collected particles that were between 3.30 µm and 8.61 µm in diameter were brightly fluorescent under UV light, but this diminished quickly in the matter of minutes. A red-orange colored filtrate remained in the stages and did not appear visibly fluorescent (Figure 2B). When the commercial green tracer dye was nebulized with the BAN system, the impactor yielded red-orange colored particles ≤3.30 µm that did not appear fluorescent under UV light (Figure 2C). With the nebulized vitamin B2 solution, aerosols ≤5.39 µm in diameter were generated, and the filtered particles were visibly fluorescent under UV light (Figure 2D). For the endonasal drilling trial, small particles ≤3.30 µm exhibiting fluorescence were generated (Figure 2E).

**Table 1.** Impactor Results from Trials Using Different Fluorescent Tracers.

	Particle Size (µm) - D_50_ at 15 L/min							
Tracer	14.1	8.61	5.39	3.30	2.08	1.36	0.98	<0.98
Saline*	**−**	**−**	**−**	+	+	+	+	+
Fluorescein*	**−**	**+**	**+**	**+**	**+**	**+**	**+**	**+**
Green water dye*	**−**	**−**	**−**	**−**	**+**	**+**	**+**	**+**
Vitamin B2 (nebulized)	**−**	**−**	**+**	+	**+**	**+**	**+**	**+**
Vitamin B2 (irrigation)	**−**	**−**	**−**	**+**	+	+	+	+

*Note.* * no visibly apparent fluorescence under UV light.

+ Denotes presence of particle aggregates.

− **No particles present at the specific impactor stage**.

## Discussion

An effective method for tracing aerosols is integral to understanding baseline risk and designing mitigation strategies for reducing the transmission risk of SARS-CoV-2. Aerosols that are ≤10 μm are of great importance due to their ability to remain airborne and travel long distances.^2^ Particles that are 5 μm or less in diameter can penetrate the lower airways, potentially contributing to an increased severity and likelihood of infection^2,13-15^ In this study, we have compared the fluorescent properties of different tracers in aerosolized form. Of the different fluorescent solutions analyzed, Vitamin B2 was the superior tracer based on fluorescence reliability across a range of aerosol sizes under 14.1 μm. Fluorescein and commercial green tracer solutions did not exhibit reliable visible fluorescence in aerosol suspension. All three tracers exhibited fluorescence in solution form and thus could be used similarly in large droplet contamination trials.

Vitamin B2 can be used as a coating agent for upper aerodigestive tract mucosa. Its fluorescent property allows for survey of aerosols and droplets generated from instrumentation of the nasal cavity or nasopharynx. COVID-19 positive patients may have high viral loads in the upper aerodigestive tract, so stratifying the aerosol and droplet risk with otolaryngology procedures is important.^16^ Vitamin B2 has significant advantages compared to other tracers in that it is inexpensive, readily available and has an established safety profile. Physiologically, riboflavin functions as an essential coenzyme in multiple redox reactions required for cellular respiration. Besides its essential biochemical functions, riboflavin has therapeutic applications when administered either orally for migraine prophylaxis and cataract risk reduction or topically for treating surgery-refractory keratoconus.^17-20^ Vitamin B2 has been utilized safely as a tracer to assess medication compliance in children. In clinical trials, the study drug can be tracked by the fluorescence of vitamin B2 excreted in the urine. It has also been used in contact contamination trials.^21-23^ There is no defined FDA upper limit of oral daily intake due to a lack of evidence of any observable adverse effects from excess riboflavin ingestion even when consuming 400 mg per day for 3 months.^24,25^ This may be due to its limited capacity for absorption by the gastrointestinal mucosa and its rapid urinary clearance.^26^

In contrast to the well-documented safety of vitamin B2, there have been multiple reports of harmful reactions to conventional tracers such as fluorescein when administered topically, intravenous, and orally. Fluorescein is a widely used fluorescent marker in ophthalmology, often administered intravenously or orally for diagnostic angiography of iris vasculature. Despite the prevalence of fluorescein use, studies have reported 5-10% of patients experiencing adverse events from intravenous fluorescein injection that include mild transient reactions such as vomiting and nausea and more severe reactions such as anaphylaxis, cardiac arrest, shock, basilar artery ischemia, and myocardial infarction.^27,28^ Topical application of fluorescein on the corneal surface is considered generally safe when used to detect corneal injury, but there have been cases of severe allergic reactions reported.^29,30^ Some have suggested requiring intradermal skin testing to determine hypersensitivity to fluorescein prior to use if an allergy is suspected, but skin testing has failed to reliably identify reactors in prospective studies.^31^ While severe adverse reactions to fluorescein may be rare, there is a lack of literature outlining the upper tolerable levels of fluorescein. This prohibits its safe use as a tracer in human trials to study aerosol and droplet dispersion. Most commercially available fluorescent dyes are not approved for use in humans; many have warnings regarding contact with the skin or mucosal surfaces.^32^

There were a few limitations to this study. Although it was easy to visually distinguish the presence or absence of fluorescent particles collected by the impactor, we did not use a spectrophotometer to quantify the specific fluorescence intensity. This study did not evaluate the duration of suspension of the nebulized fluorescent particles and the effect of different environmental conditions on surface settling times.^33^ In addition, our study included only a single form of otolaryngology instrumentation; however, the use of a high-powered drill in the nasopharynx is labeled as a high-risk aerosol-generating procedure and, therefore, adequately served as a positive control.^34^ Particles in the range of 20-100 μm were not collected by the impactor; thus, the fluorescence capability of these tracers in larger particles is yet to be investigated. Further studies involving the use of vitamin B2 as a fluorescent aerosol and droplet tracer in different otolaryngology procedures are currently underway at our institution.

There is a paucity in the literature regarding safe tracers to investigate aerosol and droplet risks in human trials. We propose vitamin B2 as a safe, inexpensive, and reliable aerosol and droplet tracer to evaluate baseline risk and mitigation measures for otolaryngology procedures. At our institution, we are conducting a project with human subjects utilizing vitamin B2 tracer to assess the aerosol and droplet risk associated with common otolaryngology clinic procedures. We encourage other groups focusing on healthcare contamination models to consider vitamin B2 as a tracer especially with regards to human trials involving aerodigestive tract mucosa.

## Conclusion

Vitamin B2 is a safe, inexpensive, and reliable aerosol and droplet tracer that can be used to evaluate baseline risk and mitigation measures for otolaryngology procedures. Its reliable suspension in small particles, longevity of its fluorescence, mucosal safety profile, and prior use in human trials illustrate its superiority as a fluorescent tracer compared to fluorescein and commercial tracer dyes. These advantages of vitamin B2 makes it an ideal tracer for healthcare contamination models.

## References

[1] van Doremalen N Bushmaker T Morris DH, et al Aerosol and surface stability of SARS-CoV-2 as compared with SARS-CoV-1. N Engl J Med. 2020;382(16):1564-1567. 32182409 10.1056/NEJMc2004973 PMC7121658

[2] Tellier R Li Y Cowling BJ Tang JW Recognition of aerosol transmission of infectious agents: a commentary. BMC Infect Dis. 2019;19(1). doi:10.1186/s12879-019-3707-y PMC6357359 30704406

[3] Chahal AM van Dewark K Gooch R Fukushima E Hudson ZM A rapidly deployable negative pressure enclosure for aerosol-generating medical procedures. MedRxiv. doi:10.1101/2020.04.14.20063958

[4] Feldman O Meir M Shavit D Idelman R Shavit I Exposure to a surrogate measure of contamination from simulated patients by emergency department personnel wearing personal protective equipment. JAMA. 2020;323(20):2091-2093. 32338711 10.1001/jama.2020.6633 PMC7186917

[5] Chen JX Workman AD Chari DA, et al Demonstration and mitigation of aerosol and particle dispersion during mastoidectomy relevant to the COVID-19 era [published online May 8, 2020]. Otol Neurotol. doi:10.1097/MAO.0000000000002765. PMC7497894 32925848

[6] Workman AD Welling DB Carter BS, et al Endonasal instrumentation and aerosolization risk in the era of COVID-19: simulation, literature review, and proposed mitigation strategies. Int Forum Allergy Rhinol. 2020;10(7). doi:10.1002/alr.22577 32243678

[7] Sharma D Rubel KE Ye MJ, et al Cadaveric simulation of endoscopic endonasal procedures: analysis of droplet splatter patterns during the COVID-19 pandemic. Otolaryngol Head Neck Surg. 2020;163(1):145-150. 32423283 10.1177/0194599820929274 PMC7240308

[8] Dramićanin M Biomedical applications of luminescence thermometry. In: Dramićanin M (ed.) Luminescence Thermometry: Methods, Material, and Applications. Elsevier Science; 2018.

[9] Landon C Garza G Nebulizer aerosol performance and patient acceptance in cystic fibrosis. Am J Respir Crit Care Med. 2016;193.

[10] AeroEclipse* II. Breath Actuated Nebulizer, https://www.oxycare.eu/out/media/AeroEclipse_II_Visual_Aid.pdf. AccessedJune 14, 2020.

[11] Next Generation Impactor (NGI). Copley Scientific, https://www.copleyscientific.com/browse/inhaler-testing/aerodynamic-particle-size-distribution-apsd/apsd-for-metered-dose-inhalers-mdis/spacers-vhcs-metered-dose-inhalers-mdis-aerodynamic-particle-size-distribution-apsd/next-generation-impactor-ngi-spacers-vhcs/. Accessed June 14, 2020.

[12] Marple VA Roberts DL Romay FJ, et al Next generation pharmaceutical impactor (a new impactor for pharmaceutical inhaler testing). Part I: design. J Aerosol Med. 2003;16(3):283-299. 14572326 10.1089/089426803769017659

[13] Memoli MJ Czajkowski L Reed S, et al Validation of the wild-type influenza A human challenge model H1N1pdMIST: an A(H1N1)pdm09 dose-finding investigational new drug study. Clin Infect Dis. 2015;60(5):693-702. 25416753 10.1093/cid/ciu924 PMC4342672

[14] Joynt GM Wu WK Understanding COVID-19: what does viral RNA load really mean? Lancet Infect Dis. 2020;20(6):635-636. 32224308 10.1016/S1473-3099(20)30237-1 PMC7118539

[15] Bustin SA Mueller R Real-time reverse transcription PCR (qRT-PCR) and its potential use in clinical diagnosis. Clin Sci (Lond). 2005;109(4):365-379. 16171460 10.1042/CS20050086

[16] Zou L Ruan F Huang M, et al SARS-CoV-2 viral load in upper respiratory specimens of infected patients. N Engl J Med. 2020;382(12):1177-1179. 32074444 10.1056/NEJMc2001737 PMC7121626

[17] Thompson DF Saluja HS Prophylaxis of migraine headaches with riboflavin: a systematic review. J Clin Pharm Ther. 2017;42(4):394-403. 28485121 10.1111/jcpt.12548

[18] Glaser TS Doss LE Shih G, et al The Association of Dietary Lutein plus Zeaxanthin and B Vitamins with cataracts in the age-related eye disease study: AREDS Report No. 37. Ophthalmology. 2015;122(7):1471-1479. 25972257 10.1016/j.ophtha.2015.04.007 PMC4485544

[19] Wollensak G Spoerl E Seiler T Riboflavin/ultraviolet-a-induced collagen crosslinking for the treatment of keratoconus. Am J Ophthalmol. 2003;135(5):620-627. 12719068 10.1016/s0002-9394(02)02220-1

[20] Goldich Y Marcovich AL Barkana Y Avni I Zadok D Safety of corneal collagen cross-linking with UV-A and riboflavin in progressive keratoconus. Cornea. 2010;29(4):409-411. 20164744 10.1097/ICO.0b013e3181bd9f8c

[21] Cluss PA Epstein LH A riboflavin tracer method for assessment of medication compliance in children. Behav Res Methods Instrum Comput. 1984;16(5):444-446.

[22] Ramanujam VM-S Anderson K Grady J Lu L-J Monitoring compliance using riboflavin (vitamin B2) as a tracer for a clinical trial. Cancer Res. 2007;67(9 suppl).

[23] Ivancic WA Nishioka MG Barnes RH Jr Hubal EC Morara M Bortnick SM Development and evaluation of a quantitative video-fluorescence imaging system and fluorescent tracer for measuring transfer of pesticide residues from surfaces to hands with repeated contacts. Ann Occup Hyg. 2004;48(6):519-532. 15298849 10.1093/annhyg/meh049

[24] Riboflavin. Health Professional Fact Sheet, https://ods.od.nih.gov/factsheets/Riboflavin-HealthProfessional/. Accessed July 10, 2020.

[25] Schoenen J Lenaerts M Bastings E High-dose riboflavin as a prophylactic treatment of migraine: results of an open pilot study. Cephalalgia. 1994;14(5):328-329. 7828189 10.1046/j.1468-2982.1994.1405328.x

[26] Institute of Medicine (US) Standing Committee on the Scientific Evaluation of Dietary Reference Intakes and its Panel on Folate, Other B Vitamins, and Choline. Dietary Reference Intakes for Thiamin, Riboflavin, Niacin, Vitamin B6, Folate, Vitamin B12, Pantothenic Acid, Biotin, and Choline. Washington, DC: National Academies Press; 1998. 23193625

[27] Marcus DF Bovino JA Williams D Adverse reactions during intravenous fluorescein angiography. Arch Ophthalmol. 1984;102(6):825. 10.1001/archopht.1984.01040030651010 6732561

[28] Gómez-Ulla F Gutiérrez C Seoane I Severe anaphylactic reaction to orally administered fluorescein. Am J Ophthalmol. 1991;112(1):94. 10.1016/s0002-9394(14)76222-1 1882930

[29] el Harrar N Idali B Moutaouakkil S, et al Choc anaphylactique par application de fluorescéine sur la conjonctive oculaire [Anaphylactic shock caused by application of fluorescein on the ocular conjunctiva]. Presse Med. 1996;25(32):1546-1547. 8952662

[30] Shahid H Salmon JF Anaphylactic response to topical fluorescein 2% eye drops: a case report. J Med Case Rep. 2010;4:27. 20181047 10.1186/1752-1947-4-27 PMC2837668

[31] Kalogeromitros DC Makris MP Rouvas A Theodossiadis PG Spanoudaki N Papaioannou D Skin testing and adverse reactions in fluorescein: a prospective study. Allergy Asthma Proc. 2007;28(4):472-476. 17883917 10.2500/aap.2007.28.3024

[32] Fluorescent Water Tracing Dye - Red or Green. Factory Direct Chemicals, https://www.factorydirectchemicals.com/products/fluorescent-water-tracing-dye. Accessed July 12, 2020.

[33] Kohanski MA Palmer JN Cohen NA Aerosol or droplet: critical definitions in the COVID-19 era [published online April 23, 2020]. Int Forum Allergy Rhinol. 2020;10.1002/alr.22591. 10.1002/alr.22591 PMC7264789 32323923

[34] American Academy of Otolaryngology–Head and Neck Surgery. Academy supports CMS, offers specific nasal policy. https://www.entnet.org/content/academy-supports-cms-offers-specific-nasal-policy. Accessed April 25, 2020.

